# Medical Wards of the Mercy Hospital

**Published:** 1863-08

**Authors:** N. S. Davis

**Affiliations:** Professor of Clinical Medicine, &c., Chicago, Ill.


### MEDICAL WARDS OF THE MERCY HOSPITAL.

### REMARKS ON DIPHTHERIA.

By N. S. DAVIS, M.D., Professor of Clinical Medicine, &c., Chicago, Ill.

Gentlemen:—The case which will illustrate the subject that will occupy our attention at the present hour, is before you. You will recognize him as a patient, to whom your attention was called eight or ten days since, on account of a sub-acute rheumatism in the ankles and tarsus, of each of the feet.

He was born in Ireland, is about 25 years of age, slightly anemic, and thin in flesh. He had nearly recovered from his rheumatic affection, and had been walking about moderately two or three days. Yesterday he began to complain some of soreness and stiffness in the fauces, with some lameness of the cervical muscles, and this morning we find him confined to his bed. If you now note his symptoms carefully, you will find the skin moderately hot; the pulse 90 per minute, soft and small; the eyes watery; the tongue covered with a whitish coat; the mucous membrane of fauces red, tumefied, and tender; the tonsils considerably swollen, and the whole inner face of each covered with a thick coat of diphtheritic exudation or membrane; and the lymphatic glands in the parotid and submaxillary regions, on both sides, considerably swollen. Thus, you have all the essential symptoms of a well-marked case of diphtheria.

If such cases as this are left to pursue their natural course, unmodified by treatment, the pulse usually increases moderately in frequency, but diminishes in force for several days. The skin continues dry and warm; the breath becomes offensive; the glands of the neck remain swollen and hard; the membranous exudation separates from the tonsils and fauces, leaving more or less irregular ulcerations, the discharge from which makes the saliva fœted, sanious, and abundant; a similar suppurative inflammation extends to the Schneiderian membrane, causing a muco-purulent discharge from the nostrils, and aiding the swelling of the tonsils in rendering the respiration rattling and sometimes difficult. In the meantime, the mental faculties become more dull, the patient being drowsy, with periods of restlessness and tossing of the extremities, and sometimes delirium. If it is tending towards an unfavorable termination, deglutition becomes more difficult, and the drink often regurgitates through the nostrils; and the pulse becomes very frequent and small; the extremities cool; the sphincters relaxed with involuntary discharges; very irregular respiration; and death. In some cases in which the general and local symptoms have progressed several days, without any unfavorable indications, the diphtheric inflammation extends suddenly to the larynx, adding the dyspnœa and cough peculiar to croup; and determining an early fatal result.

In a few instances, the diphtheritic inflammation attacks, simultaneously, the fauces and larynx, causing the peculiar symptoms of croup to be present from the beginning. Such cases are distinguished from simple pseudo-membranous croup or laryngitis, by the accompanying swelling of the glands of the neck, and of the fauces, and by the lower grade of general fever.

In a large majority of the cases of diphtheria, presenting the symptoms exhibited by the patient before you, the general febrile action continues moderate for five or six days; the patient complains much of weakness, and of difficulty or pain in swallowing; the membranous exudation gradually separates from the surface of the tonsils and fauces, leaving superficial ulcerations, and an abundant and moderately fœtid flow of saliva. The ulcerations heal in four or five days; the saliva becomes natural; all febrile symptoms disappear; and the patient becomes convalescent. In most instances, the convalescence is protracted and characterized by much weakness, and a susceptibility to renal affections, anasarca, swelling and suppuration of the lymphatic glands of the neck, and sometimes paralysis. It should have been mentioned that the urine is, in many cases, albuminous during the active progress of the diphtheritic disease; and also, that diphtheritic exudations are not limited, in all cases, to the mucous membrane of the throat, but may appear on the mucous membrane of the genital organs, or upon cut or abraded surfaces in any part of the body.

*Pathology.*—From the symptoms of this case, and the brief general description of the disease just given, it is evident that diphtheria is not a mere local inflammation, but a general disease of a febrile character, accompanied by local inflammatory processes, more particularly in the fauces and glands of the neck. To understand its pathology fully, we must consider carefully those symptoms which indicate both the condition of the blood and the properties of the solids. The general tendency to the formation of membranous or diphtheritic deposits on all inflamed or abraded surfaces, the morbid condition of the secretions, and, especially, the offensive odor of the breath and saliva, plainly indicate a morbid condition of the blood, of a septic or degenerative character. The constant tendency to ulceration, and often gangrene, in the parts affected with local inflammation,' the general feebleness of capillary circulation, the muscular debility sometimes ending in paralysis, and the dulness of the mental faculties, all point to an impairment of the elementary properties of the tissues, more especially that of vital affinity, by which all atomic and secretory changes are controlled in the living organization, and the organized structures are enabled to maintain their integrity. Hence, pathologically, we must class diphtheria with the typhoidal class of febrile diseases, in all of which there is an inherent tendency to degeneration or impairment of the properties of both solids and fluids throughout the system. Whether this impairment is caused by the introduction of some subtle poison into the blood, in the form of a contagion, infection, or miasm, which, by its presence, changes the properties of the blood, and thereby, also its relations, both chemical and vital, to the organized tissues; or whether it is caused by some occult atmospheric condition, by which the oxygen, electricity, and other ingredients of the atmosphere, fail to exert their accustomed sustaining influence on the vital properties of the living organization, cannot be satisfactorily answered in the present state of medical science. But whatever may be the immediate cause, the existence of the pathological conditions described, can hardly be doubted by any one who has attentively observed the disease at the bedside. With the diminution of vital affinity in the solids, and the progressive deterioration of the blood, there is, necessarily, general impairment of both secretion and innervation.

From these views of the pathology of diphtheria, we may deduce four well-defined and rational indications for treatment:—

1st.—To arrest the deterioration of the blood.

2d.—To improve the vital affinity and, of course, the general tonicity of the tissues.

3d.—To restore the secretory organs to their natural degree of activity.

4th.—To mitigate the violence of such local inflammations as may exist in each individual case.

To fulfil the first of these indications, the chief reliance has been placed on chlorine, bromine, and iodine, or their salts, such as the chlorates of potassa and soda. From experiments recently made with the sulphites of soda and lime, and which have been fully detailed to you in former clinics, it is rendered probable that the sulphurous acid salts will be found more efficacious in the treatment of all the diseases dependent on blood-poisoning or a septic detorioration of that fluid, than those previously mentioned. As remedies for fulfilling the second indication, we place our chief reliance on quinine, iron, and pure air. Many resort to diffusable stimulants or exhilerants, such as the various alcoholic beverages. These agents, however, spend most of their direct action on the nervous centres, while, indirectly, they depress those elementary properties of the tissues which we deem it most important to sustain. If the two first indications are effectually fulfilled, the accomplishment of the third,—namely, to restore secretion,—follows as a necessary result. But in the early stage of severe diphtheria, the dryness of the skin, the scantiness of urine, and the general febrile action is often such that much advantage may be obtained from the use of such remedies as exert a more direct influence over the more important excretory functions. For it must not be forgotten, that retained excrementitious matter may become as deteriorating to the blood, and as depressing to the properties of the tissues as the primary cause of morbid action.

Consequently, such remedies as aid in restoring a healthy activity to the organs of excretion are often indicated, both to prevent the accumulation of excrementitious matter and for facilitating the elimination of any poison that may have been imbibed as the cause of the disease. Mercurial alteratives, aided by mild diaphoretics and diuretics, will fulfil this indication more promptly and efficiently than any other means.

The means designed to act locally in combatting whatever local inflammations exist, must vary according to the extent, intensity, and stage of such inflammation in each case. In the early stage, the external applications to the swollen lymphatic glands of the neck, should be anodyne and discutient, such as an infusion of aconite leaves with muriate of ammonia dissolved in it. In the more advanced stage, when the glands remain indurated and swollen, stimulating liniments may be used: such as a combination of olive oil, oil of turpentine, and chloroform; or a mixture of camphorated soap liniment and tincture of iodine. To the inflamed surface of the fauces and tonsils, in the first stage, the local applications should be of a decidedly soothing character. All cauterizing or irritating applications in this stage, I am satisfied from close observation at the bedside, positively do more harm than good. In the latter stages, when unhealthy ulcerations or gangrene actually exists, the local applications should be anti-septic and moderately stimulant. For the first stage, I generally use nothing for the interior of the throat but the following:—

℞. Chlorate of Potassi,............................ ʒj.

Hydrochloric Acid,.......................... 20gtts.

Tincture Belladonna, ......................... ʒj.

Water, ........................................... ℥iij.

Mix. Give from half a teaspoonful to a dessert spoonful every two hours, without further dilution. The application is made much more complete and easy by swallowing it, than by any process of swabing or sponging; while the introduction of the medicine into the system constitutes one of the best means for fulfilling the first indication in the general treatment. In the latter stages, the best local application is a dilute solution of chlorate of potassa and tincture of the chloride of iron. Occasionally, an ulcerated surface may be presented of that foul character that the direct application of a strong solution of sulphate of copper, or of iodine, or of per sulphate of iron, a few times would be beneficial. But in my own practice I have not found it necessary to apply anything with a swab or sponge to the throat of a diphtheritic patient during the last four years. Having thus given briefly my views concerning the nature of diphtheria and the general principles of treatment, it only remains to prescribe for the patient before us. The disease, with him, is yet in its first stage. He is somewhat anæmic and, as already mentioned, has been recently under treatment for sub-acute rheumatism. We shall direct the following prescriptions, namely:

℞. Chlorate of Potassa,.............................. ʒiss.

Hydrochloric Acid,.............................. 20 gtts.

Tincture of Belladonna,......................... ʒiij.

Water,............................................ ℥iij.

Mix, and give a teaspoonful every two hours.

℞. Sulph. Quinine,.................................16 grs.
Pulv. Doveri,.......................................40 "
Hydrag. chlind. mite,............................ 8 "

Mix, divide into eight powders, and give one every six hours. At the same time we will keep the swollen lymphatic glands, behind the angles of the jaw, covered with a cloth wet in the following infusion, viz.:—

℞. Aconite leaves,.................................. ℥j.

Muriate of Ammonia,............................ ℥ss.

Mix; and pour on them one quart of boiling water, and use only slightly warm.

The first prescription, taken in small and frequently repeated doses, constitutes the only local application necessary for the throat; while, as an internal medicine, it is efficient in counteracting the further degeneration of the blood. The second prescription will aid in improving the tonicity or vital affinity of the solids, gently promote the excretions, and allay irritability. In about forty-eight hours we shall expect to find the white exudation on the tonsils mostly removed, and in its place some degree of ulceration. The saliva will then be more abundant and offensive; the general febrile symptoms less; the pain in swallowing less acute; but the patient complaining of much weakness. If no evacuation from the bowels occurs during that time, we shall give him a mild laxative, and substitute for the previous prescriptions the following:—

℞. Tinct. Ferri Chloridi, ............................. ℥ss.

Potassa Chlor.,............................................ ʒij.

Aqua,.......................................................... ℥iv.

Mix, and give a teaspoonful every three hours, and one dose of sulphate of quinine and Dover's powder each night and morning. If the glands of the neck remain swelled and hard, then apply, three times a day, the following liniment:—

℞. Camphorated Soap Lin.,......................... ℥ij.

Tinct. Iodine,.............................................. ℥j. Mix.

Under this treatment we shall expect the patient to be fully convalescent from the diphtheric disease in from six to eight days.

*Note.*—In the progress of the above case, the diphtheritic exudations had disappeared by the third day, leaving only slight ulcerations: and by the seventh day, the patient was quite well, except a little swelling of the glands behind the angle of the jaw, on the right side, and some general debility.

